# Supplementary material for: The extraembryonic serosa is a frontier epithelium providing the insect egg with a full-range innate immune response
Source: eLife. 2014 Dec 9;3:e04111. doi: 10.7554/eLife.04111 (PMC4358341; doi:10.7554/eLife.04111)
Supplement: Supplementary file 1. — Summary statistics for Tribolium castaneum transcriptome sequencing analysis. DOI: http://dx.doi.org/10.7554/eLife.04111.013 [file elife04111s001.docx]

**Supplementary File 1 - Summary statistics for *Tribolium castaneum* transcriptome sequencing analysis**

|  | **Wild-type** naïve | **Wild-type** Sterile  injury | **Wild-type**  Septic  injury | **Control** naïve | **Control** Sterile  injury | **Control**  Septic  injury | ***Tc-zen1*** naïve | ***Tc-zen1***  Sterile  injury | ***Tc-zen1***  Septic  injury |
| --- | --- | --- | --- | --- | --- | --- | --- | --- | --- |
| Total number of reads | 125.606.566 | 118.228.848 | 111.973.312 | 118.082.098 | 93.829.278 | 88.600.634 | 105.098.874 | 104.365.836 | 104.486.870 |
| Read length (bases) | 51 | 51 | 51 | 51 | 51 | 51 | 51 | 51 | 51 |
| Reads mapped to predicted gene models | 91.282.990 | 86.941.799 | 81.689.571 | 85.358.407 | 66.792.236 | 63.989.531 | 76.109.994 | 76.073.663 | 75.659.733 |
| Reads not mapped | 34.323.576 | 31.287.049 | 30.283.741 | 32.723.691 | 27.037.042 | 24.611.103 | 28.988.880 | 28.292.173 | 28.827.137 |
| Percentage mapped | 72.67 | 73.54 | 72.95 | 72.29 | 71.18 | 72.22 | 72.43 | 72.89 | 72.41 |
