# Supplementary material for: The extraembryonic serosa is a frontier epithelium providing the insect egg with a full-range innate immune response
Source: eLife. 2014 Dec 9;3:e04111. doi: 10.7554/eLife.04111 (PMC4358341; doi:10.7554/eLife.04111)
Supplement: Supplementary file 2. — Significantly differentially expressed immune genes in wild-type, control, and Tc-zen1 RNAi eggs. DOI: http://dx.doi.org/10.7554/eLife.04111.014 [file elife04111s002.docx]

**Supplementary File 2 - Significant differently expressed immune genes in *Tribolium castaneum* eggs**Green = induction Red = repression Black = not significant

SP = Serine protease ; SPH = Non catalytic serine protease ; cSP = Clip-domain serine protease

| **Gene ID** | **Description** | **Wildtype eggs** | | | | **Control eggs** | | | | ***Tc-zen1* eggs** | | | |
| --- | --- | --- | --- | --- | --- | --- | --- | --- | --- | --- | --- | --- | --- |
|  |  | **Sterile injury** | | **Septic injury** | | **Sterile injury** | | **Septic injury** | | **Sterile injury** | | **Septic injury** | |
|  |  | **Fold change** | **FDR**  **adjusted p-value** | **Fold change** | **FDR**  **adjusted p-value** | **Fold change** | **FDR adjusted p-value** | **Fold change** | **FDR**  **adjusted p-value** | **Fold change** | **FDR**  **adjusted p-value** | **Fold change** | **FDR adjusted p-value** |
| **Microbial recognition** | |  |  |  |  |  |  |  |  |  |  |  |  |
| TC002789 | PGRP-LA | 1.63 | 0.31 | 3.12 | **<0.01** | 2.53 | **<0.01** | 3.33 | **<0.01** | 1.12 | 1.00 | 1.67 | 1.00 |
| TC002790 | PGRP-LC | 1.28 | 1.00 | 1.65 | 0.09 | 1.88 | **<0.01** | 2.43 | **<0.01** | 1.40 | 0.57 | 1.29 | 1.00 |
| TC010611 | PGRP-SA | 4.53 | 0.96 | 241.06 | **<0.01** | 3.40 | 0.49 | 217.03 | **<0.01** | 1.11 | 1.00 | 9.38 | 0.89 |
| TC013620 | PGRP-SB | 119.59 | **<0.01** | 937.86 | **<0.01** | Inf | **<0.01** | Inf | **<0.01** | 1.92 | 0.99 | 34.74 | 0.31 |
| TC011529 | βGRP2 | 2.65 | **<0.01** | 4.94 | **<0.01** | 2.53 | **<0.01** | 5.03 | **<0.01** | 1.13 | 1.00 | 1.29 | 1.00 |
| TC003991 | βGRP3 | 4.38 | **<0.01** | 4.90 | **<0.01** | 4.22 | **<0.01** | 5.47 | **<0.01** | 2.12 | 1.00 | 1.02 | 1.00 |
| TC000808 | TEP-D | 1.29 | 1.00 | 1.18 | 1.00 | 0.64 | 0.19 | 0.57 | **0.05** | 0.71 | 0.80 | 0.70 | 1.00 |
| TC001981 | LpR2 | 1.29 | 0.44 | 1.47 | **<0.01** | 1.26 | 0.06 | 1.40 | **<0.01** | 1.40 | **0.04** | 1.41 | 0.21 |
| TC006978 | C-type lectin1 | 0.83 | 1.00 | 0.40 | **<0.01** | 0.70 | 0.08 | 0.41 | **<0.01** | 0.84 | 1.00 | 0.72 | 1.00 |
| TC003708 | C-type lectin6 | 2.16 | **<0.01** | 2.38 | **<0.01** | 2.86 | **<0.01** | 2.99 | **<0.01** | 1.20 | 0.38 | 1.16 | 0.56 |
| TC013911 | C-type lectin13 | 0.22 | **<0.01** | 0.21 | **<0.01** | 0.51 | 0.07 | 0.38 | **<0.01** | 1.52 | 1.00 | 1.64 | 1.00 |
| **Extracellular signal transduction and modulation** | |  |  |  |  |  |  |  |  |  |  |  |  |
| TC000248 | cSPH-H3 | 0.75 | 0.54 | 0.48 | **<0.01** | 0.81 | 0.41 | 0.61 | **<0.01** | 0.78 | 0.65 | 0.84 | 1.00 |
| TC000249 | cSPH-H4 | 9.50 | **<0.01** | 16.54 | **<0.01** | 7.38 | **<0.01** | 12.31 | **<0.01** | 1.13 | 1.00 | 2.00 | 1.00 |
| TC000252 | cSPH-H6 | 1.20 | 0.81 | 1.23 | 0.16 | 1.11 | 0.83 | 1.16 | 0.41 | 1.37 | **0.02** | 1.31 | 0.31 |
| TC000740 | SPH-H17 | 0.58 | **<0.01** | 0.36 | **<0.01** | 0.60 | **0.04** | 0.52 | **<0.01** | 0.60 | 0.68 | 0.86 | 1.00 |
| TC000829 | SPH-H18 | 2.87 | **<0.01** | 3.92 | **<0.01** | 2.38 | **<0.01** | 3.02 | **<0.01** | 0.62 | 1.00 | 0.90 | 1.00 |
| TC002150 | cSPH-H34 | 1.09 | 1.00 | 2.40 | 0.60 | 4.84 | 0.13 | 5.91 | **0.04** | 2.91 | 0.33 | 3.01 | 0.80 |
| TC004622 | cSPH-H51 | 1.02 | 1.00 | 1.06 | 1.00 | 0.66 | **0.03** | 0.60 | **<0.01** | 0.84 | 0.61 | 0.98 | 1.00 |
| TC004900 | SPH-H57 | 6.42 | **0.02** | 22.34 | **<0.01** | 10.36 | **<0.01** | 14.76 | **<0.01** | 4.06 | **0.02** | 3.68 | 0.23 |
| TC005908 | SPH-H64 | 20.64 | **<0.01** | 16.37 | **<0.01** | 6.54 | **<0.01** | 7.57 | **<0.01** | 3.21 | 1.00 | 1.44 | 1.00 |
| TC006246 | SPH-H70 | 252.58 | **<0.01** | 495.16 | **<0.01** | 21.54 | **<0.01** | 59.55 | **<0.01** | 3.48 | 1.00 | 5.22 | 1.00 |
| TC006247 | SPH-H71 | Inf | 1.00 | Inf | 0.13 | Inf | 0.14 | Inf | **<0.01** | 0.00 | 1.00 | 0.00 | 1.00 |
| TC007026 | cSPH-H78 | 1.16 | 1.00 | 0.81 | 1.00 | 1.59 | **<0.01** | 1.13 | 0.78 | 1.05 | 1.00 | 0.95 | 1.00 |
| TC011067 | cSPH-H125 | 1.09 | 1.00 | 1.84 | 0.14 | 2.48 | **<0.01** | 2.26 | **0.02** | 2.00 | **0.04** | 1.94 | 0.23 |
| TC000495 | cSP-P8 | 7.51 | **<0.01** | 26.66 | **<0.01** | 10.81 | **<0.01** | 40.80 | **<0.01** | 1.43 | 1.00 | 1.48 | 1.00 |
| TC000496 | SP-P9 | 36.96 | **<0.01** | 110.13 | **<0.01** | 7.17 | **<0.01** | 13.49 | **<0.01** | 0.88 | 1.00 | 1.03 | 1.00 |
| TC000497 | cSP-P10 | 4.39 | **<0.01** | 7.19 | **<0.01** | 8.02 | **<0.01** | 12.89 | **<0.01** | 1.21 | 1.00 | 1.46 | 1.00 |
| TC000547 | SP-P13 | 0.67 | **<0.01** | 0.51 | **<0.01** | 0.68 | **<0.01** | 0.51 | **<0.01** | 0.72 | **0.02** | 0.75 | 0.23 |
| TC002659 | SP-P36 | 5.44 | **<0.01** | 5.06 | **<0.01** | 4.73 | **<0.01** | 4.15 | **<0.01** | 2.16 | 0.93 | 4.42 | 0.89 |
| TC004160 | cSP-P44 | 1,43 | **<0.01** | 1.97 | **<0.01** | 1.78 | **<0.01** | 2.24 | **<0.01** | 0.99 | 1.00 | 1.04 | 1.00 |
| TC004523 | SP-P46 | 4.69 | **<0.01** | 7.19 | **<0.01** | 5.38 | **<0.01** | 7.50 | **<0.01** | 1.26 | 0.92 | 1.51 | 0.47 |
| TC004535 | SP-P50 | 3.26 | **<0.01** | 4.40 | **<0.01** | 3.69 | **<0.01** | 4.90 | **<0.01** | 0.79 | 0.97 | 0.69 | 0.94 |
| TC005976 | cSP-PSH | 2.08 | **<0.01** | 2.96 | **<0.01** | 2.54 | **<0.01** | 3.08 | **<0.01** | 1.60 | 0.41 | 1.64 | 0.80 |
| TC006033 | SP-P68 | 1.57 | **<0.01** | 1.74 | **<0.01** | 1.37 | **<0.01** | 1.48 | **<0.01** | 1.09 | 1.00 | 1.02 | 1.00 |
| TC006034 | SP-P69 | 1.38 | **<0.01** | 2.19 | **<0.01** | 1.57 | **<0.01** | 2.57 | **<0.01** | 1.52 | **<0.01** | 1.53 | **0.02** |
| TC008653 | cSP-P83 | 20.28 | **<0.01** | 17.73 | **<0.01** | 40.65 | **<0.01** | 33.53 | **<0.01** | 2.15 | 1.00 | 1.41 | 1.00 |
| TC008657 | cSP-P84 | 12.81 | 0.18 | 15.19 | **0.02** | 5.11 | 0.24 | 3.06 | 0.79 | 3.13 | 1.00 | 3.97 | 1.00 |
| TC009089 | cSP-P90 | 1.84 | 0.47 | 2.24 | **<0.01** | 1.37 | 0.74 | 2.14 | **<0.01** | 1.03 | 1.00 | 0.95 | 1.00 |
| TC009090 | cSP-P91 | 2.04 | **<0.01** | 4.23 | **<0.01** | 2.12 | **<0.01** | 5.62 | **<0.01** | 1.29 | 0.95 | 1.39 | 1.00 |
| TC009092 | cSP-P93 | 3.31 | **<0.01** | 7.32 | **<0.01** | 2.92 | **<0.01** | 7.04 | **<0.01** | 1.19 | 0.99 | 1.06 | 1.00 |
| TC009093 | cSP-P94 | 0.82 | 1.00 | 0.49 | **<0.01** | 1.16 | 1.00 | 0.72 | 0.11 | 0.72 | 1.00 | 0.68 | 1.00 |
| TC009094 | cSP-P95 | 1.50 | 0.08 | 1.36 | 0.11 | 2.35 | **<0.01** | 2.00 | **<0.01** | 1.55 | 0.39 | 1.55 | 0.87 |
| TC011078 | cSP-P126 | 7.69 | **<0.01** | 8.28 | **<0.01** | 4.11 | **<0.01** | 5.81 | **<0.01** | 0.92 | 1.00 | 1.37 | 1.00 |
| TC013277 | cSP-P136 | 4.38 | **<0.01** | 5.84 | **<0.01** | 6.27 | **<0.01** | 7.46 | **<0.01** | 1.37 | 0.61 | 1.59 | 0.37 |
| TC013326 | cSP-P140 | 1.34 | 0.07 | 1.50 | **<0.01** | 1.79 | **<0.01** | 1.82 | **<0.01** | 0.93 | 0.93 | 1.05 | 1.00 |
| TC015110 | SP-P153/SR-A4 | 1.15 | 1.00 | 1.20 | 0.81 | 1.68 | **0.04** | 1.38 | 0.32 | 0.67 | 0.53 | 0.74 | 1.00 |
| TC015295 | SP-P156 | 1.66 | 1.00 | 12.17 | **<0.01** | 2.45 | **<0.01** | 12.49 | **<0.01** | 1.66 | 0.66 | 1.54 | 0.85 |
| TC015297 | SP-P157 | 0.75 | 0.98 | 0.62 | **0.03** | 0.82 | 0.54 | 0.66 | **<0.01** | 0.48 | **<0.01** | 0.64 | 0.07 |
| TC002085 | serpin2 | 0.63 | 0.29 | 0.75 | 0.50 | 0.64 | 0.19 | 0.61 | **0.01** | 0.82 | 0.77 | 0.86 | 1.00 |
| TC005750 | serpin18 | 0.57 | **<0.01** | 0.51 | **<0.01** | 0.56 | **<0.01** | 0.56 | **<0.01** | 0.45 | **<0.01** | 0.71 | 0.21 |
| TC005751 | serpin19 | 0.76 | 0.97 | 0.97 | 1.00 | 0.73 | 0.61 | 0.94 | 1.00 | 0.32 | **0.04** | 0.43 | 0.33 |
| TC005752 | serpin20 | 0.53 | **<0.01** | 0.62 | **0.03** | 0.74 | 0.36 | 0.74 | 0.49 | 0.57 | 0.11 | 0.69 | 0.86 |
| TC005753 | serpin21 | 0.67 | 0.82 | 0.44 | **<0.01** | 0.57 | 0.13 | 0.50 | **0.01** | 0.40 | **<0.01** | 0.41 | 0.14 |
| TC005754 | serpin22 | 3.40 | **<0.01** | 4.09 | **<0.01** | 3.64 | **<0.01** | 4.32 | **<0.01** | 0.87 | 1.00 | 0.87 | 1.00 |
| TC006255 | serpin24 | 7.70 | **<0.01** | 13.68 | **<0.01** | 8.81 | **<0.01** | 13.71 | **<0.01** | 1.39 | 0.06 | 1.60 | **<0.01** |
| TC007869 | serpin26 | 6.38 | **<0.01** | 14.76 | **<0.01** | 7.56 | **<0.01** | 17.97 | **<0.01** | 1.09 | 1.00 | 1.33 | 1.00 |
| TC011718 | serpin27 | 3.00 | **<0.01** | 5.38 | **<0.01** | 3.15 | **<0.01** | 5.23 | **<0.01** | 1.03 | 1.00 | 0.90 | 1.00 |
| TC013310 | serpin28 | 2.93 | **<0.01** | 4.44 | **<0.01** | 2.82 | **<0.01** | 3.80 | **<0.01** | 1.04 | 1.00 | 1.08 | 1.00 |
| TC014237 | serpin30 | 8.42 | **<0.01** | 7.51 | **<0.01** | 5.64 | **<0.01** | 5.47 | **<0.01** | 1.04 | 1.00 | 1.22 | 1.00 |
| TC000520 | Spz1 | 1.41 | 0.42 | 1.76 | **<0.01** | 1.43 | 0.17 | 1.45 | 0.08 | 1.08 | 1.00 | 1.09 | 1.00 |
| TC001054 | Spz2 | 10.86 | **0.01** | 58.65 | **<0.01** | 12.16 | **<0.01** | 42.30 | **<0.01** | 2.64 | 1.00 | 2.32 | 1.00 |
| TC006726 | Spz4 | 0.56 | **0.02** | 0.42 | **<0.01** | 0.76 | 0.30 | 0.51 | **<0.01** | 0.71 | 0.60 | 0.81 | 1.00 |
| TC013304 | Spz5 | 0.42 | **<0.01** | 0.23 | **<0.01** | 0.61 | 0.14 | 0.35 | **<0.01** | 0.56 | 0.69 | 0.55 | 0.91 |
| **Toll-signalling pathway** | |  |  |  |  |  |  |  |  |  |  |  |  |
| TC004438 | Toll3 | 1.58 | **<0.01** | 1.96 | **<0.01** | 2.01 | **<0.01** | 2.49 | **<0.01** | 0.91 | 0.97 | 0.95 | 1.00 |
| TC000625 | Toll9 | 1.34 | 1.00 | 1.19 | 1.00 | 0.56 | 0.12 | 0.50 | **0.03** | 0.67 | 0.43 | 0.67 | 0.77 |
| TC008202 | ML1 | 0.53 | 0.46 | 0.43 | **0.02** | 0.68 | **<0.01** | 0.59 | **<0.01** | 0.64 | 0.12 | 0.90 | 1.00 |
| TC002003 | Cactus | 1.18 | 1.00 | 1.52 | 0.06 | 1.27 | 0.07 | 1.28 | **0.03** | 1.08 | 1.00 | 1.09 | 1.00 |
| **IMD-signalling pathway** | |  |  |  |  |  |  |  |  |  |  |  |  |
| TC010851 | IMD | 2.02 | **<0.01** | 3.31 | **<0.01** | 1.78 | **<0.01** | 3.39 | **<0.01** | 0.92 | 1.00 | 0.94 | 1.00 |
| TC014026 | casps4 | 0.77 | 0.70 | 0.74 | 0.21 | 0.90 | 0.98 | 0.88 | 0.79 | 0.71 | **0.02** | 0.76 | 0.13 |
| TC011191 | REL1 | 1.04 | 1.00 | 2.08 | **<0.01** | 1.06 | 1.00 | 2.39 | **<0.01** | 1.29 | **0.03** | 1.38 | **0.04** |
| TC014708 | NFAT | 0.39 | **<0.01** | 0.31 | **<0.01** | 0.39 | **<0.01** | 0.30 | **<0.01** | 0.32 | **<0.01** | 0.42 | **<0.01** |
| **JNK-signalling pathway** | |  |  |  |  |  |  |  |  |  |  |  |  |
| TC010766 | Puckered | 1.28 | 1.00 | 1.77 | 0.06 | 1.22 | 0.63 | 1.46 | **<0.01** | 1.54 | **0.04** | 1.46 | 0.40 |
| TC011870 | Kay | 1.27 | 1.00 | 1.93 | 0.08 | 1.23 | 0.28 | 1.52 | **<0.01** | 1.43 | **<0.01** | 1.49 | **0.05** |
| **Execution mechanisms** | |  |  |  |  |  |  |  |  |  |  |  |  |
| TC007737 | Attacin1 | 16.69 | 0.10 | 869.41 | **<0.01** | 56.07 | **<0.01** | 3696.08 | **<0.01** | 4.02 | 0.70 | 44.43 | 0.18 |
| TC007738 | Attacin2 | 12.25 | 0.60 | 3098.01 | **<0.01** | 6.62 | 0.21 | 2190.29 | **<0.01** | 0.47 | 1.00 | 5.28 | 1.00 |
| TC006250 | Defensin1 | 1.29 | 1.00 | 187.05 | 0.07 | 5.93 | 1.00 | 1551.17 | **<0.01** | 0.51 | 1.00 | 6.30 | 1.00 |
| TC010517 | Defensin2 | 22.67 | 0.78 | 1183.49 | **<0.01** | Inf | 0.07 | Inf | **<0.01** | 0.60 | 1.00 | 2.56 | 1.00 |
| TC012469 | Defensin3 | 16.53 | 0.24 | 907.69 | **<0.01** | Inf | 0.10 | Inf | **<0.01** | 1.84 | 1.00 | 8.26 | 1.00 |
| TC000517 | Thaumatin1 | 56.16 | **<0.01** | 89.30 | **<0.01** | 71.76 | **<0.01** | 103.03 | **<0.01** | 2.17 | 0.28 | 1.58 | 1.00 |
| TC000499 | Cecropin1 | no hit | no hit | Inf | 0.65 | no hit | no hit | Inf | **0.01** | no hit | no hit | no hit | no hit |
| TC000500 | Cecropin3 | Inf | **<0.01** | Inf | **<0.01** | 21.26 | **<0.01** | 48.59 | **<0.01** | no hit | no hit | no hit | no hit |
| TC005093 | Coleoptericin1 | 7.61 | 0.60 | 2392.39 | **<0.01** | 24.39 | **0.02** | 18067.24 | **<0.01** | 0.46 | 1.00 | 12.60 | 1.00 |
| TC005096 | Coleoptericin2 | 10.09 | 1.00 | 91.36 | 0.38 | 32.45 | 0.18 | 227.32 | **<0.01** | 1.04 | 1.00 | 7.30 | 0.91 |
| TC000325 | ProPO1 | 1.58 | 0.34 | 1.80 | **0.03** | 1.38 | 0.36 | 1.52 | **0.03** | 0.93 | 0.95 | 0.96 | 1.00 |
| TC014907 | ProPO2 | 0.84 | 1.00 | 0.72 | 0.59 | 0.74 | **<0.01** | 0.73 | **<0.01** | 0.91 | 0.63 | 0.88 | 0.71 |
| TC005377 | hexamerin4 | 13.49 | **<0.01** | 13.73 | **<0.01** | 16.05 | **<0.01** | 10.85 | **<0.01** | 22.95 | **<0.01** | 23.87 | **<0.01** |
| TC011090 | catalase2 | 0.96 | 1.00 | 0.88 | 0.99 | 0.54 | **<0.01** | 0.59 | **0.01** | 0.73 | 0.65 | 0.68 | 0.96 |
| TC010362 | Glutathione oxidase 1 | 1.47 | 0.16 | 1.79 | **<0.01** | 1.44 | 0.41 | 1.64 | **0.02** | 1.50 | 0.17 | 1.38 | 0.78 |
| TC005493 | Heme peroxidase 1 | 1.03 | 1.00 | 1.31 | 0.30 | 1.33 | 0.89 | 1.89 | **0.02** | 1.21 | 1.00 | 1.19 | 1.00 |
| TC004551 | Heme peroxidase 5 | 2.01 | **<0.01** | 2.79 | **<0.01** | 2.03 | **<0.01** | 2.74 | **<0.01** | 2.66 | **<0.01** | 2.32 | **<0.01** |
| TC000175 | Heme peroxidase 7 | 2.14 | **<0.01** | 2.25 | **<0.01** | 2.17 | **0.02** | 2.08 | **0.01** | 1.06 | 1.00 | 1.28 | 1.00 |
| TC001556 | Heme peroxidase 9 | 0.96 | 1.00 | 1.02 | 1.00 | 0.62 | **<0.01** | 0.61 | **<0.01** | 0.62 | **<0.01** | 0.62 | **<0.01** |
| TC004592 | Heme peroxidase 11 | 7.36 | **<0.01** | 12.07 | **<0.01** | 5.57 | **<0.01** | 8.83 | **<0.01** | 1.65 | 0.51 | 1.41 | 1.00 |
| TC011676 | Superoxide dismutase 2 | 1.31 | **0.03** | 1.20 | 0.21 | 1.24 | 0.24 | 1.09 | 0.80 | 0.92 | 1.00 | 0.92 | 1.00 |
| TC011675 | Superoxide dismutase 4 | 1.20 | 1.00 | 1.57 | 0.67 | 2.84 | **0.05** | 2.59 | 0.08 | 2.21 | 0.96 | 1.65 | 1.00 |
| TC010356 | Savenger receptor B13 | 3.10 | **<0.01** | 4.80 | **<0.01** | 3.33 | **<0.01** | 4.68 | **<0.01** | 6.06 | **<0.01** | 4.95 | **<0.01** |
| TC012758 | Savenger receptor B16 | 0.85 | 1.00 | 0.68 | 0.11 | 0.84 | 0.68 | 0.71 | **0.03** | 0.85 | 0.51 | 1.02 | 1.00 |
| TC015854 | Savenger receptor B2 | 1.32 | 0.16 | 1.60 | **<0.01** | 1.50 | **<0.01** | 1.86 | **<0.01** | 0.72 | 0.10 | 0.68 | 0.32 |
| TC008210 | Savenger receptor B3 | 9.30 | **<0.01** | 17.12 | **<0.01** | 24.52 | **<0.01** | 38.10 | **<0.01** | 0.66 | 1.00 | 1.13 | 1.00 |
| TC014946 | Savenger receptor B5 | 1.97 | **<0.01** | 2.77 | **<0.01** | 2.91 | **<0.01** | 3.51 | **<0.01** | 1.38 | 1.00 | 1.50 | 0.95 |
| TC000948 | Savenger receptor B6 | 1.15 | 1.00 | 0.51 | 0.21 | 0.94 | 0.98 | 0.46 | **<0.01** | 1.13 | 1.00 | 0.34 | 1.00 |
| TC014954 | Savenger receptor B9 | 0.72 | 0.17 | 0.67 | **0.02** | 0.80 | 0.12 | 0.72 | **<0.01** | 1.08 | 1.00 | 1.13 | 0.86 |
|  |  |  |  |  |  |  |  |  |  |  |  |  |  |
| TC015640 | Savenger receptor C | 1.11 | 1.00 | 1.39 | **0.04** | 1.27 | 0.79 | 1.369 | 0.37 | 1.63 | **<0.01** | 1.50 | 0.15 |
| TC011427 | Nimrod A | 0.70 | 1.00 | 0.45 | **0.03** | 0.48 | **<0.01** | 0.32 | **<0.01** | 0.49 | **0.02** | 0.55 | 0.34 |
| **Stress-related immune-responsive genes** | |  |  |  |  |  |  |  |  |  |  |  |  |
| TC015563 | apoD | 7.50 | **<0.01** | 15.03 | **<0.01** | 6.39 | **<0.01** | 12.51 | **<0.01** | 1.11 | 1.00 | 1.15 | 1.00 |
| TC010172 | Hsp68 | 6.06 | **<0.01** | 9.53 | **<0.01** | 4.86 | **<0.01** | 7.53 | **<0.01** | 2.25 | 0.23 | 1.84 | 0.86 |
| TC005338 | Hsp27 | 29.05 | 0.37 | 66.54 | 0.07 | 28.20 | **<0.01** | 67.72 | **<0.01** | 7.53 | **<0.01** | 5.02 | 0.11 |
|  |  |  |  |  |  |  |  |  |  |  |  |  |  |
| **Total number of immune genes** | | **368** | | **368** | | **368** | | **368** | | **368** | | **368** | |
| **Total upregulated** | | **45** | | **62** | | **57** | | **72** | | **13** | | **7** | |
| **Total downregulated** | | **8** | | **16** | | **10** | | **23** | | **9** | | **2** | |
