# Supplementary material for: The extraembryonic serosa is a frontier epithelium providing the insect egg with a full-range innate immune response
Source: eLife. 2014 Dec 9;3:e04111. doi: 10.7554/eLife.04111 (PMC4358341; doi:10.7554/eLife.04111)
Supplement: Supplementary file 3. — Primers used for RT-qPCR. DOI: http://dx.doi.org/10.7554/eLife.04111.015 [file elife04111s003.docx]

**Supplementary File 3 – Primers used for RT-qPCR**

| **Gene** | | **Forward primer** | | **Reverse primer** |  |
| --- | --- | --- | --- | --- | --- |
| ***Tribolium castaneum*** |  | |  | | |
| *PGRP-LC* | 5’-GAAGGAACGCTCGCTAACCC-3’ | | 5’-CGACAGCACCATCACACTCA-3’ | | |
| *SPH-H57* | 5’-ACCCATCCACAATATGCGGG-3’ | | 5’-CGCGGGATTATTTTGGTCTCC-3’ | | |
| *SPH-H70* | 5’-GACAATTTCCGTGGCAGGTG-3’ | | 5’-ACACCCACAGAATCCATTTCAT-3’ | | |
| *cSP-P8* | 5’-CCGAATGTGGAGTCCAGGAAG-3’ | | 5’-AATGAGAGTTCCGCCACAGG-3’ | | |
| *Serpin 24* | 5’-TGCCACTGCTGTCATTTTCC-3’ | | 5’-AAACCTCGGGCGAAACAACT-3’ | | |
| *Serpin 26* | 5’-GGGTGTCTGAACGGACCAAA-3’ | | 5’-AAGGGACGTCTTTTCGTGCT-3’ | | |
| *Toll3* | 5’-AACCAGATTACGGGCAACTACA-3’ | | 5’-TGCAGCTCTTGTAAGCCTATGA-3’ | | |
| *TC004646* | 5’-GCTGATCCTCGCACTGTGTA-3’ | | 5’-GCGAAAACGACGAAGAATTTCATTT-3’ | | |
| *TC007763* | 5’-AGTCTTTTGTATGCGTAGCACTC-3’ | | 5’-GTGCTGGTAGACGGGAACTG-3’ | | |
| *TC007858* | 5’-GCACATGGCGAGCCAGATTA-3’ | | 5’-GTCTCTCCCACCCACAATGG-3’ | | |
| *TC008806* | 5’-ACGCTAGTGACTGTGTGGTC-3’ | | 5’-CCAAAACTTTCCCGTTGCCT-3’ | | |
| *TC015479* | 5’-GACCCTCATCCTGTGCTGTC-3’ | | 5’-GTTGTTGCACTCCGCTTCTT-3’ | | |
